# Real-World Outcomes of Nivolumab, Pembrolizumab, and Atezolizumab Treatment Efficacy in Korean Veterans with Stage IV Non-Small-Cell Lung Cancer

**DOI:** 10.3390/cancers15164198

**Published:** 2023-08-21

**Authors:** Ahrong Ham, Young Lee, Hae Su Kim, Taekyu Lim

**Affiliations:** 1 Division of Hematology-Oncology, Department of Internal Medicine, Ewha Womans University Medical Center, Ewha Womans University College of Medicine, Seoul 07985, Republic of Korea; aham@ewha.ac.kr; 2 Division of Hematology-Oncology, Department of Internal Medicine, Veterans Health Service Medical Center, Seoul 05368, Republic of Korea; 3 Veterans Medical Research Institute, Veterans Health Service Medical Center, Seoul 05368, Republic of Korea

**Keywords:** older patients, non-small-cell lung cancer, immune checkpoint inhibitor, immunotherapy, real-world data

## Abstract

**Simple Summary:**

In clinical settings, patients receiving immune checkpoint inhibitors (ICIs) have different treatment criteria than those enrolled in clinical trials. There are concerns regarding the efficacy of ICIs in older adults due to age-associated decline in the immune system, and no study has directly compared the efficacy of different ICIs for the elderly in a real-world setting. We aimed to analyze ICIs’ use and treatment outcomes in Korean veterans with stage IV non-small-cell lung cancer (NSCLC). Three cohort groups were derived based on the ICI type (pembrolizumab, nivolumab, and atezolizumab treatment groups), and their clinical characteristics and survival outcomes were compared. There was no difference in the overall survival (OS) rate among the groups, no treatment-specific OS benefit was observed relative to the tumor PD-L1 expression, and bone metastasis was a poor prognostic factor for OS. Our study demonstrates that all three agents may be appropriate treatment options for elderly patients.

**Abstract:**

Purpose: To provide a comprehensive analysis of ICI usage and treatment outcomes in elderly Korean veterans with stage IV NSCLC. Methods: Patients diagnosed with stage IV NSCLC between 2016 and 2021 were included, and three cohorts were derived according to the type of ICI received. Thereafter, the clinical characteristics and survival outcomes were compared. Results: Of the 180 patients with NSCLC (median age, 76 years) included in this study, 49 (27.7%), 61 (33.9%), and 70 (38.9%) received pembrolizumab, nivolumab, and atezolizumab, respectively, and 19.4%, 36.1%, and 34.4% had PD-L1 expressions < 1%, 1–49%, and ≥50%, respectively. The pembrolizumab, nivolumab, and atezolizumab groups, the objective response rates (ORR), and the disease control rates (DCR) were 22.4%, 8.2%, and 4.3% (*p* = 0.004), and 59.2, 55.7%, and 30.0% (*p* = 0.001), respectively. However, no difference in the overall survival (OS) rate was noted among the groups (12.6 months vs. 8.4 months vs. 7.7 months, *p* = 0.334). Similarly, there was no treatment specific OS benefit with respect to the tumor PD-L1 expression status. Interestingly, multivariate analysis identified bone metastasis as a significant poor prognostic factor for OS (HR = 2.75 [95% CI, 1.31–5.76], *p* = 0.007). Conclusion: Pembrolizumab and nivolumab showed stronger associations with increases in ORR and DCR than atezolizumab, but no statistically significant differences were observed with respect to OS.

## 1. Introduction

**Figure 1:**
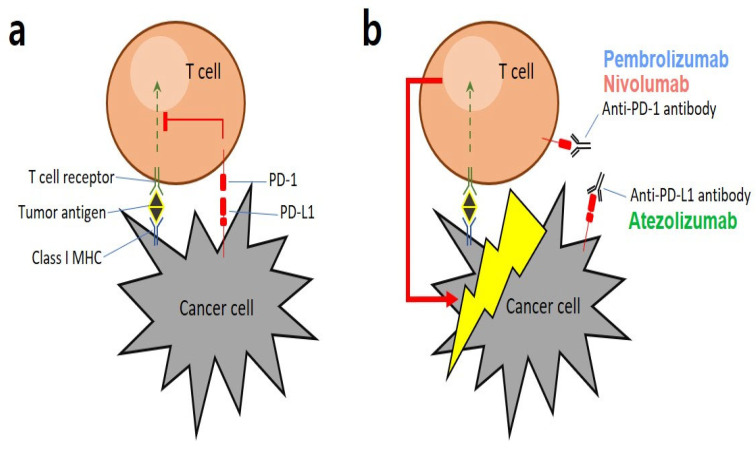
Graphical representation of key mechanisms involved in PD-1/PD-L1: (**a**) Tumor antigens are presented by major histocompatibility complexes (MHCs) that interact with antigen-specific T-cell receptors. As a strategy for escaping the immune system, cancer cells express a specific protein called PD-L1 on their surface, which binds to PD-1 present on T cells to suppress cytotoxic T cell functions. (**b**) Immune checkpoint inhibitors (ICIs), such as anti-PD-1 or anti-PD-L1 antibodies, bind to the binding sites of cancer and T cells to block immune evasion signals, allowing T cells that are not hindered by immune evasion to destroy cancer cells [3].

Lung cancer is the leading cause of cancer-related deaths worldwide, and non-small cell lung cancer (NSCLC) accounts for 82% of all lung cancer cases [1]. Treatment with immune checkpoint inhibitors (ICIs) targeting programmed death 1 receptor (PD-1)/programmed death ligand 1 (PD-L1) is a revolutionary development in oncology and has been approved for advanced or metastatic NSCLC with no epidermal growth factor receptor (EGFR) or anaplastic lymphoma kinase (ALK) genomic alterations [2,3,4]. ICIs block the immune evasion pathway used by cancer cells for survival and proliferation, among several other mechanisms, which induce immune tolerance in cancer cells (Figure 1). Pembrolizumab (KEYNOTE-010 [5]), nivolumab (CheckMate 017 and CheckMate 057 [6]), and atezolizumab (OAK [7]) are three mono-immunotherapies recommended for patients who experience cancer progression after platinum-doublet chemotherapy. Notably, according to KEYNOTE-024 results, pembrolizumab monotherapy is the standard first-line treatment for metastatic NSCLC with a PD-L1 Tumor Proportion Score (TPS) ≥ 50% [8]. Thus, ICIs are becoming increasingly recognized as conventional treatments for NSCLC [9].

Recruitment for these clinical trials was limited to younger patients with good performance status (PS; Eastern Cooperative Oncology Group [ECOG], PS score 0 or 1) and minimal comorbidities other than autoimmune diseases. Lung cancer mainly affects older adults, with approximately 70% and 30% of new cases diagnosed in patients aged ≥65 and ≥75 years, respectively [1]. The Food and Drug Administration (FDA) conducted an analysis involving older patients with lung cancer enrolled in clinical trials for drug approval; approximately 40% of these patients were aged ≥65 years, which is inconsistent with the finding that 70% of all new stage IV lung cancer cases are diagnosed in patients aged ≥65 years [10]. Therefore, the patients enrolled based on the restrictive selection criteria do not represent real-world patient populations. Consequently, the data on the relative safety and efficacy of new drugs in older patients with lung cancer and multiple comorbidities are limited.

Preliminary evidence suggests that there is little difference in the efficacy of ICIs between older and younger patients; however, the impact of ICIs on older adults remains largely unknown [11]. Furthermore, some clinical factors, such as PS and metastatic sites, have emerged as potential predictors of immunotherapy efficacy [12,13,14]. Notably, some meta-analyses and systemic reviews have been conducted to assess the efficacy and safety of different ICIs; however, only one meta-analysis has compared these three drugs. Passiglia et al. [15] performed a meta-analysis of all phases II/III randomized clinical trials (RCTs) comparing PD1/PDL1 inhibitors with docetaxel in pretreated patients with NSCLC. They indirectly compared differences in the efficacy and safety profiles of atezolizumab, pembrolizumab, and nivolumab. Based on their findings, they concluded that nivolumab and pembrolizumab were associated with a significant increase in the objective response rate (ORR) compared with atezolizumab (nivolumab vs. atezolizumab: RR 1.66, 95% confidence interval [CI] 1.07–2.58 and pembrolizumab vs. atezolizumab: RR 1.94, 95% CI, 1.30–2.90); however, no statistically significant differences were observed with respect to progression-free survival (PFS) or overall survival (OS). Regarding safety, nivolumab was found to be associated with a significantly lower risk of G3/G5 adverse events.

The Veterans Health Service (VHS) Medical Center is one of Korea’s largest integrated healthcare systems. The VHS Medical Center serves a patient population comprising individuals who tend to be older, have poorer PSs and several comorbidities, and are often underrepresented in clinical trials. Herein we report the results of a real-world observational study focused on evaluating treatment outcomes in Korean veterans with advanced NSCLC treated with three different ICI monotherapies, in first-, second-, or subsequent-line settings, at the VHS Medical Center. In addition, we investigated the predictive factors for survival outcomes in patients with NSCLC.

## 2. Methods

### 2.1. Patients and Ethics Statement

In this study, we retrospectively analyzed the data of patients with histologically confirmed clinical stage IV NSCLC at the VHS Medical Center in Korea. Patients treated with ICI monotherapy, including pembrolizumab, nivolumab, and atezolizumab, between 1 January 2016 and 31 December 2021, were included. All patients were confirmed negative for EGFR mutations and ALK gene rearrangements. Patients who received ICI combination therapy or ICI plus cytotoxic chemotherapy were excluded from the study. Metastatic or recurrent NSCLC (Stage IV) was defined radiologically using contrast-enhanced computed tomography (CT), magnetic resonance imaging (MRI), or positron emission tomography-CT (PET-CT). A total of 180 patients were enrolled and classified into three groups according to the treatment regimen. We collected data on patient demographics, including age, sex, ECOG-PS, histological subtype, line of therapy, PD-L1 expression status, metastatic sites, and survival status. This study was approved and monitored by the Institutional Review Board of the Veterans Health Service Medical Center (IRB No. 2022-07-002). The IRB waived the requirement for informed consent from the patients for this study.

### 2.2. Study Endpoint and Measures

In this study, the patients who met the inclusion criteria were divided into three groups according to the type of ICI monotherapy (pembrolizumab, nivolumab, and atezolizumab) for comparison. Response evaluation for complete and partial responses or disease progression was evaluated according to the Response Evaluation Criteria in Solid Tumors (RECIST) version 1.1 [16] based on CT imaging. The ORR was defined as the proportion of patients with complete or partial responses, whereas the disease control rate (DCR) was defined as the proportion of patients with complete, partial, or stable responses. Further, the OS was defined as the date from the start of treatment with the first ICI to the date of death from any cause. Survival was determined using the date of the last follow-up visit for patients who were alive at the time of the analysis. Furthermore, to investigate the association between PD-L1 expression and survival outcome, the entire cohort of patients was divided into three groups according to the PD-L1 expression status (PD-L1 < 1%, 1–49%, and ≥50%), and OS was further analyzed according to the PD-L1 expression level. Measurements, including survival outcomes and prognostic factors for survival, were performed until treatment discontinuation, loss of follow-up, death, or the last follow-up.

### 2.3. Statistical Analysis

All statistical analyses were performed using the statistical software SPSS version 25 (IBM, Chicago, IL, USA) and R software version 4.1.2 (R Development Core Team, Vienna, Austria). Patient demographics were analyzed using Pearson’s chi-square or Fisher’s exact test for categorical variables, and the analysis of variance or the Kruskal–Wallis test for continuous variables. The median OS was estimated using the Kaplan–Meier method with log-rank tests. Patient survival was monitored until 23 August 2022. Univariate and multivariate analyses were also performed using Cox proportional-hazards regression models to identify factors associated with survival outcomes in patients with NSCLC. Hazard ratios (HR) and 95% CIs were estimated by adjusting for age, sex, ECOG-PS, histological subtype, PD-L1 expression level, treatment, and metastasis sites. For the multivariate analysis, the above-mentioned variables were analyzed using backward selection (stopping condition: *p* < 0.15). Statistical significance was set at *p* < 0.05.

## 3. Results

### 3.1. Patient Characteristics

A total of 180 patients with recurrent or metastatic NSCLC, who received ICI monotherapy were recruited. The median age of all patients was 76 years (interquartile range, 74–78 years), and 177 patients (98.3%) were male (Table 1). Notably, most patients with NSCLC had an ECOG-PS score of 0 or 1 (96.1%) at the start of the treatment, whereas the remaining 3.9% had an ECOG-PS score of 2 or 3. Furthermore, all the patients had a current or past smoking history, and among them, 49 (27.7%), 61 (33.9%), and 70 (38.9%) were categorized into the pembrolizumab, nivolumab, and atezolizumab groups, respectively. The squamous cell histological subtype was the most common NSCLC subtype in the atezolizumab group, accounting for 60.0% of cases (squamous: pembrolizumab, n = 18 [36.7%]; nivolumab, n = 30 [49.2%]; and atezolizumab, n = 42 [60.0%]), whereas the non-squamous cell histological subtype was the least common in this group (non-squamous: pembrolizumab, n = 31 [63.3%]; nivolumab, n = 31 [50.8%]; and atezolizumab, n = 28 [40.0%]). Only 8 of the 180 patients (4.4%) in the pembrolizumab group received ICI as first-line therapy, whereas the remaining 172 (95.6%) received ICI as second-line therapy and beyond. Of all patients with known tumor PD-L1 expression statuses, 19.4%, 36.1%, and 34.4% had PD-L1 < 1%, 1–49%, and ≥50%, respectively. The most common metastatic sites were the brain (n = 21, 11.7%), bones (n = 13, 7.2%), and liver (n = 8, 4.4%).

### 3.2. Treatment Outcomes

**Figure 2:**
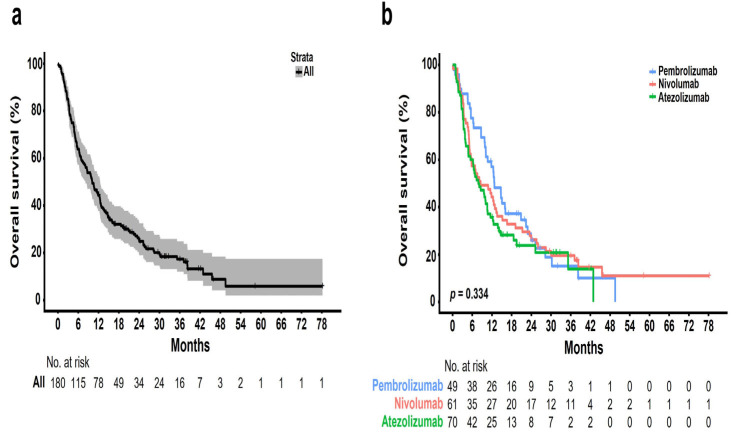
Kaplan–Meier plots of overall survival (OS): (**a**) entire cohort of patients; (**b**) patients stratified according to different ICIs.

**Figure 3:**
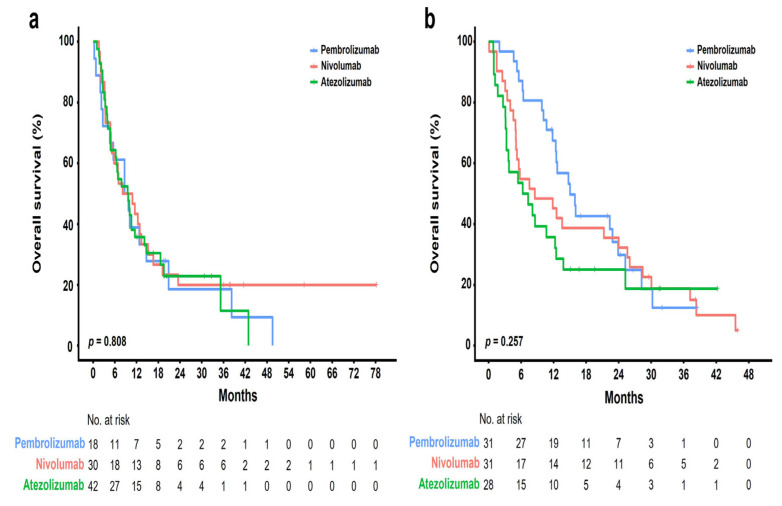
Kaplan–Meier plots of OS according to different ICIs in patients with squamous and non-squamous histological subtypes: (**a**) squamous; (**b**) non-squamous.

**Figure 4:**
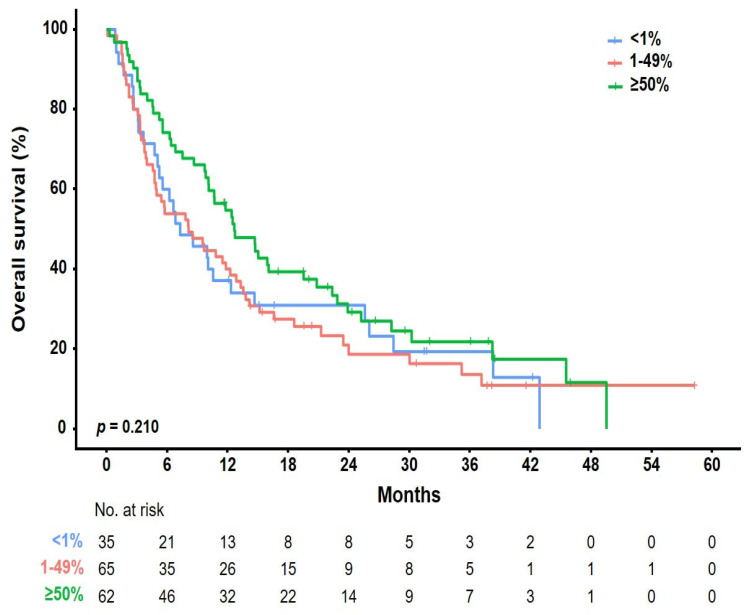
Kaplan–Meier plots of OS according to PD-L1 expression status. Data of 162 patients with confirmed PD-L1 expressions were analyzed. Three different PD-L1 diagnostic assays were included: the PD-L1 IHC 22C3 pharmDx, Ventana PD-L1 SP263, and Ventana PD-L1 (SP142) assays. The most important results were based on several PD-L1 assays performed on patients individually.

The median follow-up period was 10 months (range, 1–77 months), and the median number of cycles was four (range, 1–123). The ORR was 22.4% [95% CI, 11.77–36.62], 8.2% [95% CI, 2.72–18.10], and 4.3% [95% CI, 0.89–12.02] in the pembrolizumab, nivolumab, and atezolizumab groups, respectively (Table 2). Further, the DCR was 59.2% [95% CI, 44.21–73.00], 55.7% [95% CI, 42.45–68.45], and 30.0% [95% CI, 19.62–42.13] in the pembrolizumab, nivolumab, and atezolizumab groups, respectively. None of the groups showed a complete response. Partial response was achieved in 11 (22.4%), 5 (8.2%), and 3 (4.3%) patients who received pembrolizumab, nivolumab, and atezolizumab, respectively. Further, stable disease was achieved in 18 (36.7%), 29 (47.5%), and 18 (25.7%) patients who received pembrolizumab, nivolumab, and atezolizumab, respectively. The median OS was 10 months [95% CI, 7.9–12.0] (Figure 2a). There was no significant difference in the OS rate among the treatment groups (pembrolizumab [12.6 months] vs. nivolumab [8.4 months] vs. atezolizumab [7.7 months], *p* = 0.334; Figure 2b). Figure 3 shows the OS stratified according to the histological subtype. In the squamous cell histological subtype, the median OS was 8.5, 8.0, and 9.4 months in the pembrolizumab, nivolumab, and atezolizumab groups, respectively (Figure 3a). For the non-squamous cell histological subtype, the median OS was 14.8, 8.4, and 6.2 months in the pembrolizumab, nivolumab, and atezolizumab groups, respectively (Figure 3b). The statistical significance of OS according to the histological subtype was not confirmed (squamous, *p* = 0.808 and non-squamous, *p* = 0.257), and the OS for the pembrolizumab and nivolumab groups was shorter for patients with the squamous histological subtype than for those with the non-squamous histological subtype. Among 162 patients (90.0%) with a confirmed tumor PD-L1 expression, the OS rate according to the PD-L1 expression showed no significant difference (*p* = 0.210) (Figure 4). The median OS was 7.2 months, 8.0 months, and 12.5 months in the PD-L1 < 1%, 1–49%, and ≥50% groups, respectively (<1% vs. 1–49%, *p* = 0.805; 1–49% vs. ≥50%, *p* = 0.101; and <1% vs. ≥50%, *p* = 0.203). Regarding tumor PD-L1 expression, there was no treatment-specific OS benefit in the PD-L1 < 1%, 1–49%, and ≥50% expression groups (Figure S1a–c).

### 3.3. Prognostic Factors Affecting Overall Survival

The univariate and multivariate Cox proportional hazards models for identifying the prognostic factors affecting the OS of patients with NSCLC are described in Table 3. No factors were independently associated with poor OS in the univariate analysis. In the multivariate analysis, bone metastasis was identified as a significantly poor prognostic factor for OS (bone metastasis: HR = 2.75 [95% CI, 1.31–5.76], *p* = 0.007).

### 3.4. Safety Outcomes

Treatment-related adverse events were reported in 48 patients, 50% of whom experienced immune-related adverse events (irAEs) (Table 4). The most common irAEs were skin rash (5.5%), pneumonitis (3.3%), and thyroid dysfunction (3.3%). Grade ≥ 3 irAEs were reported in one patient in the pembrolizumab group and two patients in the atezolizumab group, respectively. There was no death related to irAEs.

## 4. Discussion

This study is the first in which real-world data analysis of the efficacy of nivolumab, pembrolizumab, and atezolizumab, approved by the FDA for the treatment of advanced NSCLC, was performed. These drugs are widely used in clinical practice. However, to the best of our knowledge, their efficacy as monotherapies in older patients in a real-world setting has never been directly compared.

The results obtained showed a lower ORR, as well as a similar median OS relative to previously published RCTs (ORR: 4.3–22%; overall, 10.6%; DCR: 30.0–59.2%; overall, 46.7%) (Table 5). Age-related decline in the immune system, known as “immunosenescence”, may affect immune checkpoint suppression activity in older patients [17,18]. In addition, many comorbidities may contribute to death from causes other than lung cancer in older adults [19,20]. Notably, several real-world studies on older patients have shown similar results. However, it is unreasonable to compare the efficacy of immunotherapy between the present study and the existing RCTs based on age. For an accurate comparison, many other parameters such as tumor histology, PD-L1 score, treatment regimen, and line of therapy must be corrected. Therefore, this study aimed to compare and analyze treatment efficacy and OS within the study cohort rather than directly comparing it with existing RCTs.

We classified a consecutive cohort of patients with advanced stage IV NSCLC into three groups according to the ICI monotherapy regimen. The three groups comprised older adults with an average age ≥ 75. Some vulnerable patients with an ECOG-PS score ≥ 2 were also included. Regarding efficacy, the ORRs for the pembrolizumab, nivolumab, and atezolizumab groups were 22.4%, 8.2%, and 4.3%, respectively, indicating that pembrolizumab and nivolumab were more beneficial than atezolizumab. Further, Nivolumab significantly improved ORR over atezolizumab based on correspondence analysis (nivolumab vs. atezolizumab, *p* = 0.006; pembrolizumab vs. atezolizumab, *p* = 0.471; and pembrolizumab vs. nivolumab, *p* = 0.066). Similarly, the DCR was better improved in the pembrolizumab (59.2%) and nivolumab (55.7%) groups than in the atezolizumab (30.0%) group. Similar results were also observed following concordance analysis (nivolumab vs. atezolizumab, *p* = 0.003; pembrolizumab vs. atezolizumab, *p* = 0.005; and pembrolizumab vs. nivolumab, *p* = 0.866). However, we could not conclude that pembrolizumab and nivolumab were more effective than atezolizumab in terms of efficacy. Several reasons could account for this observation. First, a squamous histology was more common for patients who received atezolizumab than those who received other drugs. In this study, the PD-L1 expression rate was high in the non-squamous histology. Therefore, patients with a non-squamous histology with these features were treated with either pembrolizumab or nivolumab. As reported in several pivotal RCTs [6] and the real-world outcome literature [23,24,25], the squamous histological subtype tends to be associated with shorter PFS and OS than the non-squamous histology. Second, in this study, we did not treat each drug evenly according to the PD-L1 expression status. Based on the results of the existing pivotal RCTs, in the present study, most patients with high PD-L1 expression levels were treated with pembrolizumab and nivolumab, whereas atezolizumab was mainly used in patients with a low or unconfirmed PD-L1 expression status. Therefore, patients in the atezolizumab group may show a relatively poor efficacy. Despite these conditions, there were no differences in OS among the three groups (12.6, 8.4, and 7.7 months for pembrolizumab, nivolumab, and atezolizumab groups, respectively), with pembrolizumab showing the longest OS duration. However, this difference was not statistically significant (*p* = 0.334). Additionally, we confirmed the OS of patients receiving ICIs according to PD-L1 expression status via correspondence analysis. In the PD-L1 1–49% subgroup, when the OS according to ICIs was analyzed in correspondence, no statistical significance was observed (pembrolizumab vs. atezolizumab, *p* = 0.092; nivolumab vs. atezolizumab, *p* = 0.911; and pembrolizumab vs. nivolumab, *p* = 0.053) (Figure S1b). In contrast, in the PD-L1 ≥ 50% subgroup, pembrolizumab showed a statistically significant OS benefit compared with the atezolizumab (pembrolizumab vs. atezolizumab, *p* = 0.023; nivolumab vs. atezolizumab, *p* = 0.153; and pembrolizumab vs. nivolumab, *p* = 0.406) (Figure S1a). Further, in the pembrolizumab group, eight patients treated with pembrolizumab as the first-line treatment were included, and there were only five patients with a PD-L1 expression ≥ 50% in the atezolizumab group. Therefore, more samples are needed to determine statistical significance.

Older patients are likely to be vulnerable to the side effects of cytotoxic chemotherapy; therefore, they receive many ICIs that are known to have relatively tolerable side effects [26]. However, the eligibility criteria for clinical trials are not representative of the patient population in real-world practice; most clinical trials include patients receiving conventional cytotoxic chemotherapy as controls. Therefore, the data on direct efficacy comparisons between ICIs are lacking.

A noteworthy finding of the present study was that bone metastases negatively affected the OS. Based on cancer biology, bone is a hematopoietic organ that is involved in the production of regulatory T, memory T and B, and cytotoxic T cells that control the immune system [27,28]. Therefore, pathological bone loss may impair the production of immune-related cells. The association between bone metastasis and survival has not been extensively studied; however, a prospective cohort study in Italy showed poor outcomes in terms of bone metastasis and survival rates [29]. In addition, several published retrospective studies have reported poor prognosis associated with bone metastasis [24,30]. Therefore, the results of this study are consistent with those of previous studies, indicating that bone metastases adversely affect OS in patients receiving ICIs. Additional treatment methods or adjuvant agents that can enhance the efficacy of ICI in patients with bone metastasis need to be investigated.

This study had several limitations. First, this is a retrospective, single-center observational study. Thus, differences in clinical baseline characteristics may have affected the efficacy evaluation. Pembrolizumab was used on the frontline and was administered more frequently to patients with a high PD-L1 expression. Therefore, these characteristics may have improved survival in the pembrolizumab group. Second, because various detection techniques are used to identify PD-L1 expression, the consistency between methods may be inaccurate. In the present study, three assays were used: PD-L1 22C3 pharmDx, VENTANA PD-L1 SP263, and VENTANA PD-L1 SP142 immunohistochemistry assays. PD-L1 expression was classified based on the highest value among the three test results. Notably, not all three techniques were performed in all patients; therefore, unifying them into one technique is impossible. However, several studies have indicated that the PD-L1 22C3 and SP263 techniques are highly consistent [31,32]. In the present study, none of the patients had their SP142 results reflected in the final result of PD-L1 expression. In addition, when only patients who underwent PD-L1 22C3 were analyzed for the accuracy of the results, there was no difference in OS according to ICI, as observed in previous studies. Third, comorbidities were not considered in this survival study. Meserve et al. [33] reported that in patients with pre-existing inflammatory bowel diseases (IBD) that were treated with ICIs, approximately 40% experienced IBD relapse and ICI discontinuation. Immune-checkpoint inhibitors should be used with caution as they may increase the risk of reactivation of an existing autoimmune disease. Furthermore, in the case of older adults, comorbidities may affect survival; however, since the study was based on the data from electronic medical records, information on comorbidities or the association between comorbidities and mortality could be overlooked. Despite these limitations, the survival outcomes in the present study were similar to those reported in existing pivotal RCTs. Similarly, this study had inherent limitations in identifying all adverse events due to the retrospective nature of the analysis, particularly due to the incomplete or missing data. However, several reports have mentioned that the frequency of immune-related adverse events is similar or lower in elderly patients than in younger patients [10,18]. Therefore, for safety reasons, there is no need to limit the use of ICIs.

## 5. Conclusions

This study is clinically significant because no previous study has directly compared the efficacy of the three different ICIs in older patients receiving ICI monotherapy. There were no statistically significant differences in survival outcomes among the three ICIs, demonstrating that they could possibly constitute an appropriate treatment option for older patients. Our results can alleviate clinicians’ concerns about deciding the type of ICI single agent and may assist in making decisions based on the patient’s outpatient visit frequency or the clinician’s preference. In addition, bone metastasis was found to be associated with poor survival outcomes after immunotherapy. Future studies are needed to determine immunotherapeutic agents with different mechanisms that are more effective according to the metastatic sites in elderly patients with lung cancer receiving monotherapy.

## Figures and Tables

**Table 1:** Patients’ baseline clinical characteristics.

		Pembrolizumab (n = 49)	Nivolumab (n = 61)	Atezolizumab (n = 70)	Total (n = 180)	*p*-Value
Age	Median (IQR)	75 (74–78)	76 (74–78)	76 (74–78)	76 (74–78)	0.635 †
Sex	Male	48 (98.0%)	60 (98.4%)	69 (98.6%)	177 (98.3%)	0.967 *
	Female	1 (2.0%)	1 (1.6%)	1 (1.4%)	3 (1.7%)	
ECOG-PS	0 or 1	46 (93.9%)	60 (98.4%)	67 (95.7%)	173 (96.1%)	0.437 *
	2 or 3	3 (6.1%)	1 (1.6%)	3 (4.3%)	7 (3.9%)	
Smoking	Current/Ex-smoker	49 (100%)	61 (100%)	70 (100%)	180 (100%)	
Histology	Squamous	18 (36.7%)	30 (49.2%)	42 (60.0%)	90 (50.0%)	0.046
	Non-squamous	31 (63.3%)	31 (50.8%)	28 (40.0%)	90 (50.0%)	
Line of therapy	1st line	8 (16.3%)	0 (0%)	0 (0%)	8 (4.4%)	<0.001 *
	2nd line	21 (42.9%)	26 (42.6%)	39 (55.7%)	86 (47.8%)	
	≥3rd line	20 (40.8%)	35 (57.4%)	31 (44.3%)	86 (47.8%)	
PD-L1 expression §	<1%	0 (0%)	10 (16.4%)	25 (35.7%)	35 (19.4%)	<0.001
	1–49%	2 (4.1%)	34 (55.7%)	29 (41.4%)	65 (36.1%)	
	≥50%	46 (93.9%)	11 (18.0%)	5 (7.1%)	62 (34.4%)	
	Unknown ‡	1 (2.0%)	6 (9.8%)	11 (15.7%)	18 (10.0%)	
Metastatic sites	Liver metastasis	6 (6.1%)	2 (3.3%)	3 (4.3%)	8 (4.4%)	0.816 *
	Brain metastasis	8 (16.3%)	10 (16.4%)	3 (4.3%)	21 (11.7%)	0.048 *
	Bone metastasis	4 (8.2%)	4 (6.6%)	5 (7.1%)	13 (7.2%)	1.000 *
Survival status	Ongoing	10 (20.4%)	9 (14.8%)	15 (21.4%)	34 (18.9%)	0.592
	Death	39 (79.6%)	52 (85.2%)	55 (78.6%)	146 (81.1%)	

* The *p*-value was calculated using Fisher’s exact test. † The *p*-value was calculated using Kruskall–Wallis test. § PD-L1 expression was analyzed using PD-L1 22C3 pharmDx assay, VENTANA PD-L1 SP263, and VENTANA PD-L1 SP142 immunohistochemistry assay. It was classified based on the highest value among the three test results. ‡ Patients with “Unknown” PD-L1 expressions have not been performed in the real world. Abbreviations: IQR, interquartile range.

**Table 2:** Summary of response rates.

Response n (%) [95% CI]	Pembrolizumab (n = 49)	Nivolumab (n = 61)	Atezolizumab (n = 70)	Total (n = 180)
**CR**	0	0	0	0
**PR**	11 (22.4) [11.77–36.62]	5 (8.2) [2.72–18.10]	3 (4.3) [0.89–12.02]	19 (10.6) [6.48–15.99]
**SD**	18 (36.7) [23.42–51.71]	29 (47.5) [34.60–60.73]	18 (25.7) [16.01–37.56]	65 (36.1) [29.10–43.59]
**PD**	20 (40.8) [27.00–55.79]	22 (36.1) [24.16–49.37]	48 (68.6) [56.37–79.15]	90 (50.0) [42.47–57.53]
**NA §**	0	5 (8.2) [2.72–18.10]	1 (1.4) [0.04–7.70]	6 (3.3) [1.23–7.11]
**ORR**	11 (22.4) [11.77–36.62]	5 (8.2) [2.72–18.10]	3 (4.3) [0.89–12.02]	19 (10.6) [6.48–15.99]
**DCR**	29 (59.2) [44.21–73.00]	34 (55.7) [42.45–68.45]	21 (30.0) [19.62–42.13]	84 (46.7) [39.21–52.24]

§ Not assessable indicates patients who discontinued treatment before the first response evaluation without evidence of progressive disease or who did not undergo post-baseline imaging after treatment. Abbreviations: CI, confidence interval; CR, complete response; PR, partial response; SD, stable disease; PD, progressive disease; NA, not assessable; ORR, objective response rate; DCR, disease control rate.

**Table 3:** Prognostic factors for overall survival based on uni- and multi-variate Cox proportional hazards models.

		Event/Total ‡ (146/180)	Univariate HR (95% CI)	Univariate *p*-Value	Multivariate HR § (95% CI)	Multivariate *p*-Value
Age		146/180	1.01 (0.98–1.04)	0.369		
Gender	Male	143/177	1			
	Female	3/3	1.70 (0.54–5.36)	0.365		
ECOG-PS	0 or 1	140/173	1			
	2 or 3	6/7	0.92 (0.40–2.08)	0.834		
Histology	Squamous	73/90	1			
	Non-squamous	73/90	0.90 (0.65–1.24)	0.528		
PD-L1	<1%	29/35	1			
	1–49%	54/65	1.04 (0.66–1.64)	0.859	1.12 (0.71–1.77)	0.635
	≥50%	48/62	0.76 (0.48–1.20)	0.241	0.73 (0.46–1.16)	0.179
	Missing †	15/18				
Treatment	Atezolizumab	55/70	1			
	Pembrolizumab	39/49	0.75 (0.50–1.14)	0.179		
	Nivolumab	52/61	0.84 (0.57–1.23)	0.361		
Liver meta	No	141/172	1			
	Yes	5/8	0.90 (0.37–2.21)	0.826		
Brain meta	No	130/159	1			
	Yes	16/21	0.97 (0.58–1.64)	0.915	0.61 (0.32–1.17)	0.136
Bone meta	No	135/167	1			
	Yes	11/13	1.83 (0.99–3.39)	0.056	2.75 (1.31–5.76)	0.007

‡ Number of patients included in the univariate analysis. † There are many missing values for the ‘PD-L1 expression’ variable, and patients with unconfirmed results were excluded. § A total of 162 patients were analyzed for the multivariate analysis. Missing values for the ‘PD-L1 expression’ variable were not included in the multivariate analysis. Furthermore, age and gender were excluded from the analysis because only the elderly were included in this study and there were only three women. For the multivariate analysis, the mentioned variables were analyzed using backward selection (stopping condition: *p* < 0.15). Statistical significance was set at *p* < 0.05. Abbreviations: HR, hazard ratio; CI, confidence interval.

**Table 4:** Adverse events in the safety population.

n of Events (%)	Pembrolizumab (n = 49)		Nivolumab (n = 61)		Atezolizumab (n = 70)	
	Any Grade	Grade ≥ 3	Any Grade	Grade ≥ 3	Any Grade	Grade ≥ 3
Fatigue	4 (8.2)	0	2 (3.3)	0	5 (7.1)	0
Anorexia	3 (6.1)	0	6 (8.2)	0	3 (4.2)	0
Oral mucositis	0	0	1 (1.6)	0	0	0
Diarrhea *	0	0	0	0	1 (1.4)	0
Skin rash *	5 (10.2)	0	3 (4.9)	0	2 (2.8)	0
Pneumonitis *	1 (2.0)	1 (2.0)	1 (1.6)	0	4 (5.7)	2 (2.8)
Hepatotoxicity *	1 (2.0)	0	0	0	0	0
Thyroid dysfunction *	2 (4.1)	0	2 (3.3)	0	2 (2.9)	0

* Immune-related adverse events.

**Table 5:** Characteristics and outcomes of the pivotal randomized clinical trials and the present study.

Study	Agents	Line	Histology %	Patients n	Age, Median (Range)	ECOG ≥ 2 n (%)	PD-L1 Score (%)	PFS (Months)	OS (Months)	ORR (%)
KN-010	Pembro	≥2	Sq: 22 Non-sq: 70 Other: 3	344	63 (56–69)	3 (1)	≥50	3.9	14.9	21.2
KN-024	Pembro	1	Sq: 18.8 Non-sq: 81.2	154	65 (33–90)	0	≥50	10.3	30	20.7
KN-042 [21]	Pembro	1	Sq: 38 Non-sq: 62	637	63 (57–69)	0	≥1	7.1	20	27.3
CM 017	Nivo	≥2	Squamous	135	62 (39–85)	2 (1.5)	≥10	3.5	9.2	20
CM 057	Nivo	≥2	Non-sq	292	61 (37–84)	0	≥10	2.3	12.2	19
OAK	Atezo	≥2	Sq: 26 Non-sq: 74	425	63 (33–82)	0	All-comer	2.8	13.8	14
IMpower 110 [22]	Atezo	1	Sq: 25 Non-sq: 75	107	64 (33–79)	0	≥50 or (IC ≥ 10)	8.1	20	40.2
Current study	Overall	≥1	Sq: 50 Non-Sq: 50	180	76 (74–78)	7 (3.9)	All-comer	nr	10	10.6
	Pembro Nivo Atezo			49 61 70	75 (74–78) 76 (74–78) 76 (74–78)	3 (6.1) 1 (1.6) 3 (4.3)			12.6 8.4 7.7	22.4 8.2 4.3

Abbreviations: ECOG, Eastern Cooperative Oncology Group; PFS, progression-free survival; OS, overall survival; ORR, objective response rate; nr, not reported; Pembro, pembrolizumab; Nivo, nivolumab; Atezo, atezolizumab; Sq, squamous; Non-sq, non-squamous.

## Data Availability

Data are available on reasonable request. Anonymized individual participant data and study documents can be requested from the corresponding author of this study (T.L., imegene@naver.com) upon reasonable request.

## Supplementary Materials

The following supporting information can be downloaded at: https://www.mdpi.com/article/10.3390/cancers15164198/s1. Figure S1: Comparison of survival outcomes according to different ICIs: (a) PD-L1 < 1%; (b) PD-L1 1–49%; and (c) PD-L1 ≥ 50%. Data of 162 patients with confirmed PD-L1 expressions were analyzed. No patient was treated with pembrolizumab in the PD-L1 < 1% subgroup.
