# Supplementary material for: Real-World Outcomes of Nivolumab, Pembrolizumab, and Atezolizumab Treatment Efficacy in Korean Veterans with Stage IV Non-Small-Cell Lung Cancer
Source: Cancers (Basel). 2023 Aug 21;15(16):4198. doi: 10.3390/cancers15164198 (PMC10453652; doi:10.3390/cancers15164198)
Supplement: Supplementary file 1 [file cancers-15-04198-s001.zip › cancers-2539950-supplementary.pdf]

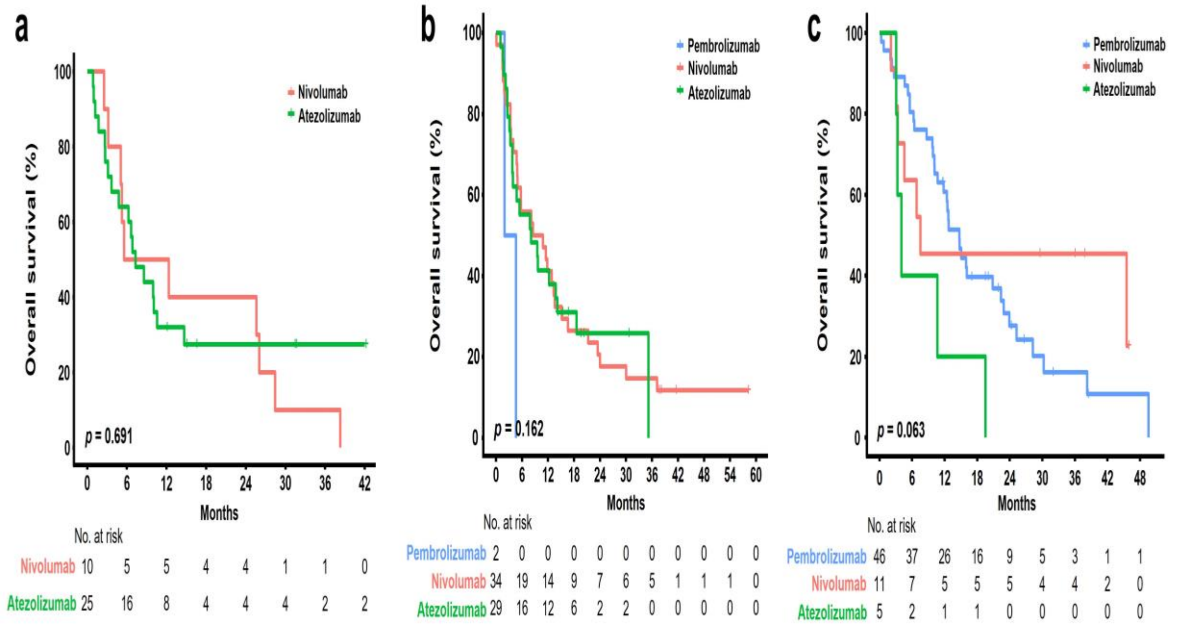

Figure S1. Comparison of survival outcomes according to different ICIs: (a) PD-L1 < 1%; (b) PD-L1 1–49%; and (c) PD-L1 ≥ 50%. Data of 162 patients with confirmed PD-L1 expressions were analyzed. No patient was treated with pembrolizumab in the PD-L1 < 1% subgroup.
